# Respiratory Morbidity in Office Workers in a Water-Damaged Building

**DOI:** 10.1289/ehp.7559

**Published:** 2005-01-20

**Authors:** Jean M. Cox-Ganser, Sandra K. White, Rebecca Jones, Ken Hilsbos, Eileen Storey, Paul L. Enright, Carol Y. Rao, Kathleen Kreiss

**Affiliations:** ^1^Centers for Disease Control and Prevention, National Institute for Occupational Safety and Health, Morgantown, West Virginia, USA; ^2^University of Connecticut Health Center, Farmington, Connecticut, USA

**Keywords:** building-related symptoms, hypersensitivity pneumonitis, indoor environment, occupational asthma, office workers, quality of life, sarcoidosis, sick leave

## Abstract

We conducted a study on building-related respiratory disease and associated social impact in an office building with water incursions in the northeastern United States. An initial questionnaire had 67% participation (888/1,327). Compared with the U.S. adult population, prevalence ratios were 2.2–2.5 for wheezing, lifetime asthma, and current asthma, 3.3 for adult-onset asthma, and 3.4 for symptoms improving away from work (*p* < 0.05). Two-thirds (66/103) of the adult-onset asthma arose after occupancy, with an incidence rate of 1.9/1,000 person-years before building occupancy and 14.5/1,000 person-years after building occupancy. We conducted a second survey on 140 respiratory cases, 63 subjects with fewer symptoms, and 44 comparison subjects. Health-related quality of life decreased with increasing severity of respiratory symptoms and in those with work-related symptoms. Symptom status was not associated with job satisfaction or how often jobs required hard work. Respiratory health problems accounted for one-third of sick leave, and respiratory cases with work-related symptoms had more respiratory sick days than those without work-related symptoms (9.4 vs. 2.4 days/year; *p* < 0.01). Abnormal lung function and/or breathing medication use was found in 67% of respiratory cases, in 38% of participants with fewer symptoms, and in 11% of the comparison group (*p* < 0.01), with similar results in never-smokers. Postoccupancy-onset asthma was associated with less atopy than preoccupancy-onset asthma. Occupancy of the water-damaged building was associated with onset and exacerbation of respiratory conditions, confirmed by objective medical tests. The morbidity and lost work time burdened both employees and employers.

As part of a program to study occupational respiratory disease in the nonindustrial environment, we investigated building-related respiratory health in the employees of a large 20-story office building in the northeastern United States. Since the mid-1990s, the building had leaked through the roof, around windows, and through sliding doors of terraces. The upper floors had suffered the most water damage and mold contamination. During investigation of these problems, the building was found to be operating at a negative pressure with respect to the outdoors, which could exacerbate water incursion through the building envelope. Furthermore, there had been plumbing leaks on many floors which had damaged interior walls. The first major construction activity related to water incursion began in 2000, with repair of roof copings and brick caulking. From 2000 to 2002, cubicle partitions and carpets were cleaned, wetted carpet and stained wallboard replaced, wallpaper and underlying mold removed from bathrooms, upgrades to the air handling system made, and windows caulked. In 2002, permanent repairs on the building exterior, including roof replacement, began to prevent water incursion.

Building occupants had reported health conditions that they considered building related. Symptom onset spanned several years, with an increase in symptoms and frequency of complaints beginning in the fall of 2000. Sentinel cases of postoccupancy-onset asthma, hypersensitivity pneumonitis (HP), and sarcoidosis had been diagnosed, and the persons affected had been relocated to another facility. HP is an immune-regulated granulomatous disease that has been associated with fungal contamination, and it has been found to coexist with asthma in damp office buildings (Arnow et al. 1978; Kreiss 1989; Hoffman et al. 1993; Jarvis and Morey 2001). Sarcoidosis is an immune-regulated granulomatous disease of unknown etiology.

In this article we report evidence of excesses of respiratory symptoms and physician diagnosis of asthma in the occupants of the water-damaged building as well as verification of self-reported respiratory illness with objective testing. We also describe the burden of illness in terms of absences, use of breathing medications, and health-related quality of life.

## Methods and Materials

### Study design and population.

In September 2001, we offered a questionnaire to all 1,327 employees working in the building. The questionnaire was administered to groups of approximately 50 employees at a time, using schedules prepared by management. During each group session, National Institute for Occupational Safety and Health (NIOSH) staff described the purpose of the survey and the consent process and read the questions aloud from overhead transparencies as the participants completed them. By completing the questionnaire, the participants were indicating consent to take part in the survey. The questionnaire comprised sections on demographics; symptoms (upper and lower respiratory, systemic, headache, and difficulty concentrating) in the last 4 weeks and 12 months, and in relation to being in the building; physician diagnosis of asthma, HP, and sarcoidosis, with dates of diagnosis; smoking history; and work history in the building. The completed questionnaires were electronically scanned into a database and hand-checked for quality control.

We used the September questionnaire to identify a group of employees who had worked in the building for at least 1 year and who met either an epidemiologic case definition for lower respiratory illness or a comparison group definition. The respiratory case definition was three or more of five lower respiratory symptoms (wheeze/whistling in the chest, chest tightness, shortness of breath, coughing, awakening by attack of breathing difficulty) occurring weekly over the past month; or at least two of three symptoms consistent with HP (shortness of breath when hurrying on level ground or walking up a slight hill, fever and chills, flulike achiness or achy joints) occurring weekly over the past month; or current asthma with postoccupancy physician diagnosis, or physician-diagnosed HP or sarcoidosis. The comparison group definition was none of the respiratory case lower respiratory or HP-like symptoms in the past year and none of the respiratory case diagnoses.

We invited the 202 employees who met the case definition and the 154 employees who met the comparison group definition to participate in a questionnaire and medical testing survey in June 2002. During the site visit, an additional 15 employees asked to take part in the survey. All participants provided written informed consent (approved by the NIOSH Human Subjects Review Board). We used results of the June questionnaire to reclassify participants into the respiratory case or comparison groups. Participants who reported lower respiratory or systemic symptoms but who did not meet the criteria of a respiratory case formed a third, “fewer symptoms” group.

### Questionnaire.

Participants completed an interviewer-administered computer-based questionnaire. The June 2002 questionnaire included sections on demographics, work history, health and symptom history, physician diagnoses, smoking, home environment, and job stress and satisfaction as used in the U.S. Environmental Protection Agency (EPA) Building Assessment Survey and Evaluation (BASE) study (U.S. EPA 1994), and health-related quality of life from the SF-12 (Medical Outcomes Study, Short Form; Ware et al. 1996). We included questions on the use of beta-agonist and corticosteroid inhalers, over-the-counter breathing medications, and other asthma medications in the previous 4 weeks, as well as oral corticosteroid use in the previous 12 months, adapted from an asthma-severity score module (Blanc et al. 1996). To help with recall, participants were asked to bring to their testing session a list of the medications that they were taking for breathing problems.

### Spirometry.

Qualified technicians followed standard guidelines for spirometry (American Thoracic Society 1995). We compared the test results to expected values for a healthy, non-smoking person of the same age, height, sex, and race using spirometry reference values and 95% normal confidence intervals (CIs) generated from the third National Health and Nutrition Examination Survey (NHANES III) (Hankinson et al. 1999). Abnormal test results were categorized as having a pattern of obstruction, restriction, or a “mixed” pattern of both airways obstruction and a low forced vital capacity (FVC) (American Thoracic Society 1995). We defined airways obstruction as a low forced expiratory volume in 1 sec (FEV_1_) to FVC ratio (FEV_1_/FVC%) with low FEV_1_. We defined restriction as a low FVC and normal FEV_1_/FVC%.

### Methacholine challenge testing.

To detect bronchial hyperresponsiveness (BHR), we performed methacholine challenge testing (MCT) using standardized techniques (Crapo et al. 2000) with 0.125, 0.5, 2, 8, and 32 mg/mL methacholine. Five breaths of nebulized methacholine were administered for each dose, with FEV_1_ measured 30 and 90 sec later. If FEV_1_ dropped > 20% of the baseline value, no further methacholine was given. We report methacholine dose as PC_20_, which is the provocative concentration of methacholine that causes an interpolated 20% decline in FEV_1_ from the baseline. We defined BHR as a PC_20_ of ≤4.0 mg/mL, and borderline BHR as a PC_20_ between 4.1 and 16.0 mg/mL (Crapo et al. 2000).

### Bronchodilator testing.

In subjects with baseline FEV_1_ < 70% of the predicted value, MCT was not performed, but a bronchodilator test was performed to detect any reversible bronchoconstriction. Two puffs of a beta-agonist were administered via metered dose inhaler and were followed by spirometry. We defined reversibility as a 12% and 200 mL FEV_1_ improvement after bronchodilator administration (American Thoracic Society 1991).

### Allergen skin prick testing.

We applied extracts of seven common indoor and outdoor allergens and three mold mixes using the GreerPIK system (Greer Laboratories, Lenoir, NC): dust mite mix (*Dermatophagoides farinae* and *D. pteronyssinus*), German cockroach (*Blattella germanica*), cat hair, seven grass mix, ragweed mix, common weed mix, Eastern eight tree mix, *Dematiaceae* mix (outdoor molds: *Alternaria tenuis, Cladosporium herbarum, Helminthosporium sativum, Pullularia pullulans, Spondylocladium atrovirens, Curvularia spicifera), Aspergillus mix*, and *Penicillium mix*. The negative control was 50% glycerin in water, and histamine served as a positive control. For each wheal, the mean diameter (average of the length and width) at 15 min was calculated. We defined a positive reaction as an average diameter at least 3 mm larger than the negative control and > 25% of the average diameter of the positive control. For the purposes of this study, atopy was defined as at least one positive skin test on allergy testing, using a total of seven common antigen extracts (excluding the mold mixes).

### Data analysis.

We compared the prevalence rates of respiratory symptoms and self-reported medical diagnoses observed in the building occupants during the September 2001 survey to the U.S. adult prevalence rates obtained from NHANES III [National Center for Health Statistics (NCHS) 1996], the 2001 data for Connecticut from the Behavioral Risk Factor Surveillance System (BRFSS) (National Center for Chronic Disease Prevention and Health Promotion Behavioral Risk Factor Surveillance System 2001), and data for occupants of 41 office buildings with no known indoor environmental problems (Apte et al. 2000). For comparisons with NHANES III, we used indirect standardization for race (black, Hispanic, white), sex, age (17–39 years of age versus 40–69 years of age), and cigarette smoking status (current, former, or never smoker). For comparisons with BRFSS, we standardized for sex. We derived 95% CIs using a method that assumes that the observed data are from a Poisson distribution (Kahn 1989).

To estimate incidence density rates of physician-diagnosed adult-onset asthma, for each participant we calculated person-time at risk for two time periods: from 16 years of age to building occupancy and from building occupancy to the September 2001 survey date. For subjects with physician-diagnosed adult-onset asthma, time at risk ended on the date of diagnosis. Time at risk for each participant was summed to give person-years at risk. Participants with childhood asthma did not contribute any time at risk.

We used SAS software (version 8.02; SAS Institute Inc. Cary, NC) to analyze the data. Chi-square tests were used in statistical analysis of two-way classification tables. We used the Cochran-Mantel-Haenszel test in analysis of differences between proportions after adjustment for smoking, and we used the Cochran-Armitage test in analysis for linear trends in proportions. We used the SAS GLM procedure to model number of days lost and Duncan’s multiple range test for multiple means comparisons.

## Results

### September 2001 Survey

#### Participation.

Participation was 67% (888/1,327) in the cross-sectional questionnaire study. Participants were predominantly white, in their mid-forties, former or never smokers, who had been working in the building for about 6 years (Table 1). We had demographic and participation information on the 689 employees working for one of the two building tenant organizations. These employees had a mean age of 45 years, and 74% were white, 19% were black, and 53% were female. There was 76% participation among these employees. Comparison between participants and nonparticipants showed no differences in mean age or race. There were proportionately more females among participants than among nonparticipants (57% vs. 40%, *p* < 0.01).

#### Excess respiratory symptoms and physician-diagnosed asthma.

In comparisons with the U.S. adult population, prevalence ratios ranged from 2.2 to 2.5 for wheezing, lifetime asthma, and current asthma (*p* < 0.05; Table 2). Nasal and eye symptoms were more prevalent in the building occupants than lower-respiratory symptoms, but were less elevated compared to U.S. adults (prevalence ratios 1.5 and 1.6, respectively, *p* < 0.05). The building occupants reported wheeze, nasal, or eye symptoms that improved when they were away from work at 3.4 times the rate of the U.S. population (*p* < 0.05). Compared to the state adult population, prevalence ratios were 1.4 (95% CI, 1.2–1.6) for lifetime asthma, and 1.5 (95% CI, 1.3–1.9) for current asthma. A majority (60–70%) of participants with wheeze, chest tightness, shortness of breath, or cough in the last 4 weeks reported an improvement in symptoms when away from the building. Prevalence ratios for work-related lower respiratory symptoms compared to U.S. office workers were elevated and ranged from 2.7 to 4.7 (*p* < 0.05; Table 3).

#### Adult onset asthma prevalence and incidence.

The prevalence of adult-onset asthma was 12% (103/865). A comparison to the U.S. adult population gave a prevalence ratio of 3.3 (95%, CI 2.7–4.0). Two-thirds (66/103) of the adult-onset asthma occurred after occupancy of the building. An analysis of adult-onset asthma incidence density was conducted based on 19,173 person-years at risk before building occupancy and 4,564 person-years at risk after building occupancy. We found incidences of 1.9 per 1,000 person-years in the period before building occupancy and 14.5 per 1,000 person-years in the period after building occupancy. The incidence rate ratio was 7.5, indicating a large increase in asthma incidence in the period after building occupancy.

#### Asthma symptom severity and exacerbation.

The participants with postoccupancy-onset, physician-diagnosed asthma had a higher mean value for the sum of cough, wheeze, chest tightness, and shortness of breath occurring once or more per week in the last 4 weeks than any other participants (*p* < 0.05). There was also a significant trend (*p* < 0.01) in prevalence of lower respiratory symptoms that improved when away from the building: 52% of those with postoccupancy-onset asthma, 41% of those with adult preoccupancy-onset asthma, 27% of those with childhood asthma, and 23% of those with no physician-diagnosed asthma (Table 4).

#### HP and sarcoidosis.

Eight participants reported HP, five with postoccupancy-onset and one with preoccupancy-onset HP (two people did not give diagnosis dates). Sarcoidosis was reported by six participants, three with postoccupancy onset, and two with preoccupancy onset (one person did not give a date of diagnosis). Fever and chills were reported as occurring once or more in the last 4 weeks by 9%, flulike achiness or achy joints by 22%, and excessive fatigue by 29% of participants. A work-related pattern was noted by 22% of those with fever and chills, by 30% of those with flulike achiness or achy joints, and by 52% of those with excessive fatigue.

### June 2002 Survey

#### Participation.

There were 248 participants in the June 2002 survey. Participation was higher among the invited employees meeting the respiratory case definition in September 2001 (142/202; 70%) than among the comparison group invitees (91/154; 59%). Based on the June 2002 questionnaire results, there were 140 participants in the respiratory case group, 63 participants in the fewer symptoms group, and 44 participants in the comparison group. One participant had missing questionnaire information and could not be classified. A little more than half of those asymptomatic in September 2001 reported symptoms 9 months later, with 17% achieving respiratory case status, and 38% falling into the fewer symptoms group. In contrast, a majority (81%) of those meeting the respiratory case definition in September 2001 still met this definition 9 months later, 17% fell into the intermediate group, and 2% became asymptomatic. The demographics of the June 2002 participants stratified by respiratory status are given in Table 5. There were more females and more current smokers in the respiratory case group.

#### Lung function tests, breathing medication use, and reported respiratory health.

Respiratory cases had the highest proportions of abnormal breathing tests and breathing medication use; the fewer symptoms group had the next highest; and the comparison group had the lowest proportions of these two outcomes (Tables 6 and 7). Test results indicated more obstruction than restriction, and the respiratory cases had a trend for a higher prevalence of obstruction than the other participants. BHR was higher in the two groups with symptoms than in the comparison group, but this finding was not significantly different. We found very little breathing medication use reported by the comparison group as compared to almost half of the respiratory cases. Analyses on the never-smokers showed similar trends, with a prevalence of abnormal lung function tests and medication use combined of 71% for respiratory cases, 30% for participants with fewer symptoms, and 12% for the comparison group.

#### Quality of life.

**Figure 1:**
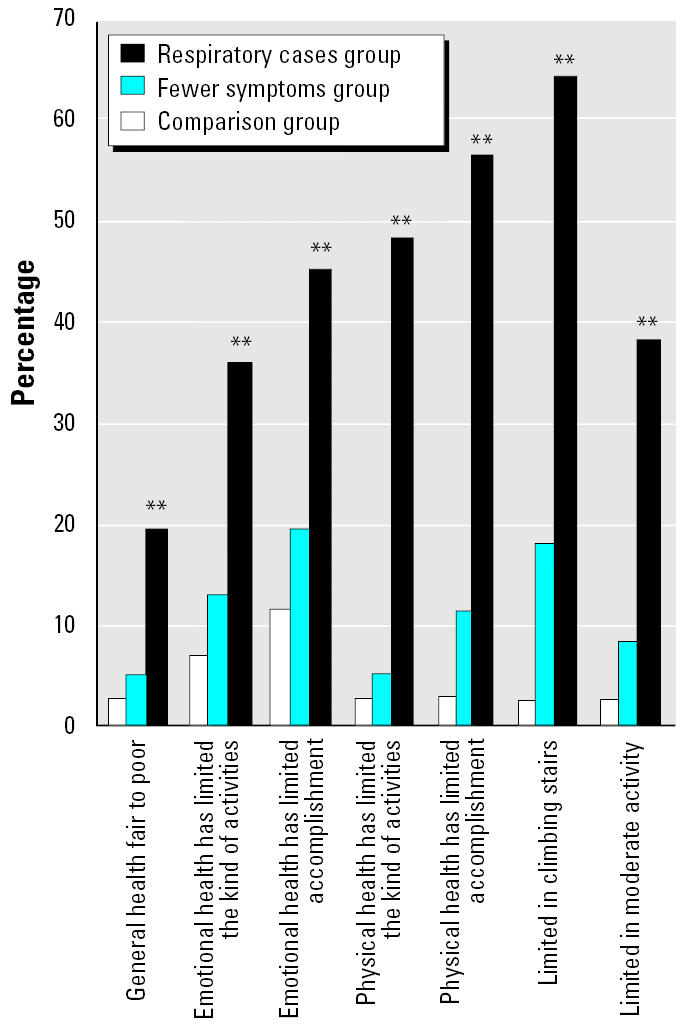
Quality-of-life comparisons among symptom groups.
**p < 0.01, Cochran-Armitage trend test.

**Figure 2:**
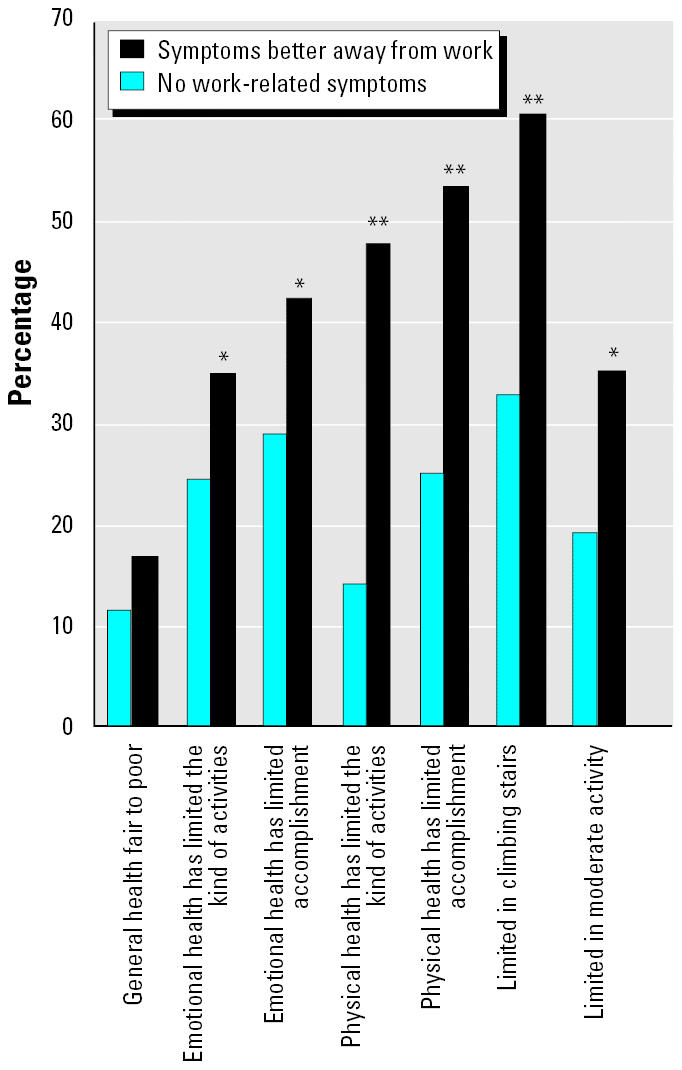
Quality of life in the respiratory case group and the fewer symptoms group, stratified by work-relatedness of symptoms.
*p < 0.05 and **p < 0.01, Chi-square test.

We compared responses to health-related quality-of-life questions among the three symptom status groups. We found statistically significant trends for increasing impairment in health-related quality of life with increasing severity of respiratory symptoms. The largest differences were seen for reported physical limitations (Figure 1). Within the respiratory case and the fewer symptoms groups, we found statistically significant poorer health-related quality of life in relation to the presence of work-related symptoms, except for general health status (Figure 2). Similar results were found for health-related quality of life and postoccupancy symptom onset, except that statistical differences were seen for limitations in climbing stairs, physical health-limiting accomplishments, and physical health limiting the kind of activities.

#### Job stress/dissatisfaction.

There were no statistical differences among symptom status groups for responses on job satisfaction or how often a person was required to work hard. Being very or somewhat satisfied with their job was reported by 87% of respiratory cases, 90% of the group with fewer symptoms and 93% of the comparison group. Being required to work hard frequently or very often was reported by 51% of respiratory cases, 62% of the intermediate group, and 45% of the comparison group.

#### Work days lost.

The number of days off work in the last 12 months due to respiratory problems was significantly associated with symptom status (*p* < 0.01). The respiratory cases had missed a mean of 6.9 days as compared to 1.7 days for the group with fewer symptoms and 2.0 days for the asymptomatic group. We found that 34% of respiratory cases had ≥6 days of respiratory sick leave, compared to 11% of the fewer symptoms group and 4.7% of the asymptomatic comparison group (*p* < 0.01). In contrast, there was no statistically significant difference between the three groups for nonrespiratory sick leave. The respiratory cases lost a mean of 4.5 days, the group with fewer symptoms lost 7.5 days, and the asymptomatic group lost 4.1 days due to nonrespiratory conditions.

The number of respiratory sick days was similar for symptomatic participants regardless of whether the onset was pre- or post-occupancy. A large effect was seen for having respiratory symptoms that improved away from the building. Respiratory cases with work-related respiratory symptoms had more respiratory sick days than those with symptoms that did not improve away from the building (9.4 vs. 2.4, *p* < 0.01). In the group with fewer symptoms, those with work-related respiratory symptoms had more respiratory sick leave than those with symptoms with no work-related pattern (3.7 vs. 1.1, *p* < 0.05).

We estimated sick days over the past year for respiratory conditions and total sick leave for building occupants by applying the mean work days missed for the three symptom groups to the number of participants in those categories from the September 2001 questionnaire (816 participants had adequate data). Respiratory health problems accounted for 34% of sick leave days (2,490/7,402). The respiratory case group represented 25% of the September 2001 participants but contributed 56% (1,401/2,490) of the respiratory sick leave days. Using the mean of 2 days of respiratory sick leave reported by the comparison group as a non–building-related baseline for building occupants gives an estimated 858 days of excess respiratory sick leave (2,490–1,632). Thus, up to 12% (858/7,402) of the preceding 12 months of employee sick leave days might have been attributable to building-related effects.

#### Breathing medication use.

We looked at the prevalence of the use of asthma-controller medications (inhaled corticosteroids, cromolyn, nedocromil, oral antileukotrienes) and reliever medications (short-acting beta-agonists and ipratropium bromide) in the last 4 weeks in participants with physician-diagnosed asthma for comparison with a national sample of 1,788 U.S. adults with current asthma (Adams et al. 2002; Fuhlbrigge et al. 2002) using two-sample tests of proportions. Use of an asthma controller was reported by 39% of our study group versus 21% of U.S. asthma cases overall (*p* < 0.01). The prevalence of 39% asthma-controller use was marginally higher (*p* = 0.07) than the value of 29% reported for U.S. cases with severe persistent symptoms in the last 4 weeks. Reliever use was reported by 50% of our group versus 63% of U.S. cases (*p* < 0.05).

#### Skin prick allergy tests.

**Figure 3:**
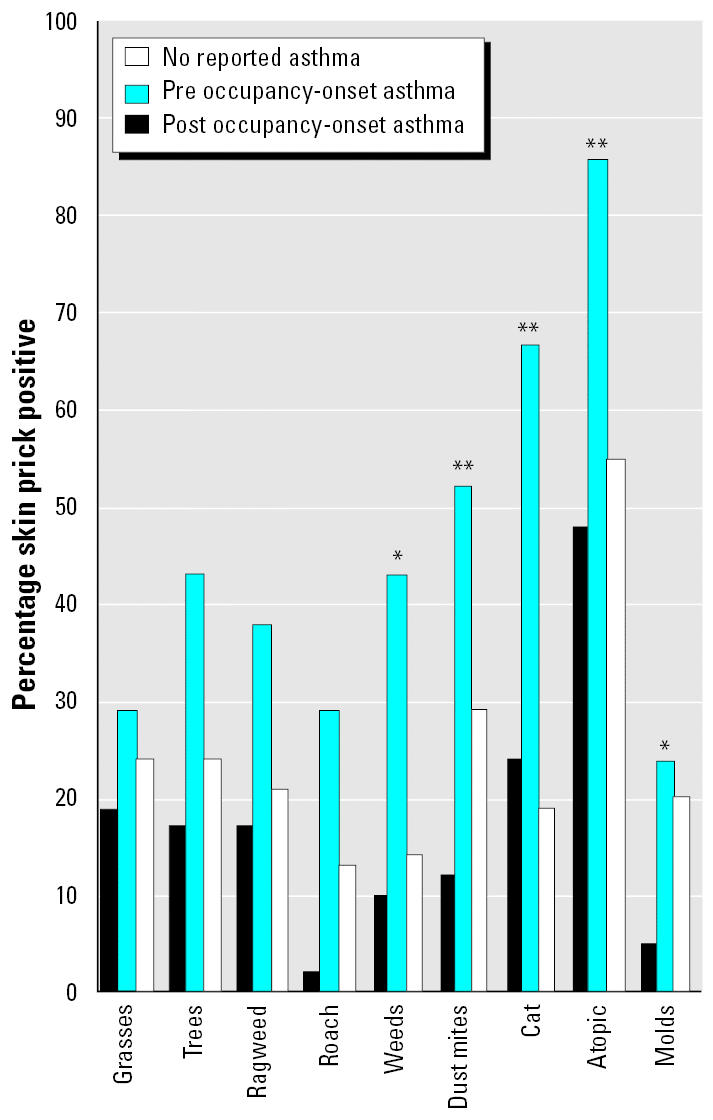
Allergy skin test results by asthma diagnosis.
*p < 0.05 and **p < 0.01, Chi-square test.

Over half of the participants met the definition for atopy. There was no statistical difference in the prevalence of atopy among the respiratory case group, the group with fewer symptoms, and the comparison group. However, preoccupancy-onset asthma was associated with a higher prevalence of atopy (*p* < 0.05). The results of individual skin prick allergen tests showed that persons with preoccupancy-onset asthma had a higher prevalence of positive reactions to cat, dust mites, and weed mix (*p* < 0.01) as well as to cockroach allergens (*p* < 0.05). We found that the postoccupancy-onset asthma cases had the lowest reaction to the mold mixes (*p* = 0.05; Figure 3).

## Discussion

Physician-diagnosed asthma and respiratory symptoms occurred in excess among our study participants and was confirmed by an excessive rate of airway obstruction and BHR. Studies of building occupants with known health concerns are subject to reporting bias. In our study, in addition to reported symptoms and physician diagnoses, we examined measures of respiratory disease, including medication use and medical tests. Two-thirds of those classified as respiratory cases based on symptoms or physician diagnoses had objective pulmonary function abnormalities or used prescription medications for breathing difficulties (given with the goal of normalizing lung function). The higher rate of lung function abnormalities and breathing medication use in the participants reporting respiratory symptoms validates the symptom reports.

The majority (60–70%) of participants with respiratory symptoms reported a work-related pattern, implying a building-related exposure. The 7% overall prevalence of work-related wheeze was higher than the 2–4% in studies of nonproblem buildings (Apte et al. 2000) and higher than the 2–6% found in studies of problem buildings (Malkin et al. 1996). In the 9-month interval between the building-wide questionnaire survey and the nested case–control survey, more than half (55%) of the comparison group chosen because they had no lower respiratory or systemic symptoms in September 2001 had become symptomatic, including 17% who were classified as respiratory cases. Improvement was rare in September 2001 cases (17%), suggesting a continued effect of building occupancy on respiratory health. Some of this response pattern may be attributable to overreporting due to general concern about water incursions and sentinel cases with health effects, but such concerns had been present since before the September 2001 survey.

The estimated incidence of physician-diagnosed, adult-onset asthma among the study participants (1.9 per 1,000 person-years) before building occupancy was within the range of other estimates for adults, for example, 2.1 per 1,000 person-years (McWhorter et al. 1989), 3.8 per 1,000 person-years (Sama et al. 2003), and about 1 per 1,000 person-years (Reed 1999). In contrast, after building occupancy, incidence rose 7.5 times to 14.5 per 1,000 person-years, consistent with the symptoms that developed in the previously asymptomatic comparison group.

The burden of respiratory problems in this population was reflected in substantial respiratory sick leave attributable to building occupancy (estimated at 12% of total). The presence of work-related respiratory symptoms was positively associated with respiratory sick leave, but time of symptom onset was not, suggesting that having a work-related pattern to respiratory symptoms was a larger determinant of respiratory sick leave than whether the symptoms arose before or after building occupancy.

The proportion of our study respiratory cases with ≥6 days of respiratory sick leave was 34%. In comparison, a population study of 1,788 adults with asthma in the United States found that 11% of participants had ≥6 days of sick leave in the past year related to their asthma (Fuhlbrigge et al. 2002). In our study, respiratory cases had a mean of 6.9 respiratory sick days, compared to 4.4 annual work absences because of breathing problems among Canadian asthmatics (Ungar and Coyte 2000). In the Canadian study more productivity was lost due to a decrease in level of functioning at work on days when breathing problems were worse than usual than due to days off work. Although we have no estimate of productivity loss due to a decrease in functioning at work for our study participants, the high prevalence of work-related symptom exacerbation suggests a substantial decrease in productivity might have occurred. High respiratory morbidity was also indicated by the high use of asthma-controller medication and the decreased prevalence of quick-relief medications. This pattern of medication use is consistent with persistent asthma associated with daily work-related exacerbation.

We found strong associations between respiratory symptom status and lower health-related quality of life, confirming the social burden of respiratory morbidity in building occupants. In contrast, we found no relation between job stress, job satisfaction, or perceived work burdens with symptom status; this is consistent with the findings of another investigation of building-related respiratory disease (Jarvis and Morey 2001) and reduces the likelihood that disgruntled employees in a problem building exaggerate their symptoms.

The specific etiology and mechanisms of the respiratory disease in this building remain undefined. The skin prick test results for immediate hypersensitivity responses to common aeroallergens were unexpected. Preoccupancy-onset asthma was associated with atopy, as anticipated [National Asthma Education and Prevention Program (NAEPP) 1997; Peden 2000]. However, postoccupancy-onset asthma cases had much lower prevalence of IgE-mediated allergen skin-test positivity (atopy). Perhaps the airway inflammation was not driven by an IgE mechanism. It is possible that nonbiologic irritant exposures were present, and furthermore, although molds have allergenic properties (Lander et al. 2001), the development of asthma in damp/moldy conditions may not be IgE mediated (Douwes et al. 2003; Savilahti et al. 2001).

The rarity of clusters of HP in the general population points to a work-related etiology for the cluster in the building occupants. The recent Institute of Medicine report on damp indoor spaces and health found sufficient evidence for an association between mold or other agents in damp indoor environments and upper respiratory tract symptoms, cough, wheeze, asthma symptoms in sensitized persons, and HP in susceptible persons (Institute of Medicine 2004). The cluster of sarcoidosis raises concern that this granulomatous lung disease was misdiagnosed HP (Forst and Abraham 1993) or has overlapping environmental causes (Kucera et al. 2003).

The major limitation of the present study is the possible influence of participation bias. We had a 67% participation in our September 2001 survey, and differences in health status of participants and nonparticipants could have led to overestimation of symptom and asthma prevalence, particularly since women were more likely to be participants. Using the most conservative approach, we compared minimum possible prevalences among the entire building population to the external reference populations. We still found excesses of asthma and symptoms in comparison to the U.S. population and to office workers in buildings not known to have indoor environmental problems (data not shown), but we found no differences in asthma prevalence in comparison to the state population. Counterbalancing possible response bias among those occupants who participated in our study is our finding of gradients of nonsubjective tests and reported medication use in relation to symptom intensity.

In conclusion, the present study contributes to the growing literature that water-damaged buildings can be associated with work-related respiratory disease. This investigation documents the considerable respiratory illness, adverse effects on quality of life, and absenteeism that have placed personal, social, and economic burdens on many employees and their employers. Building-related respiratory disease warrants increased public health, medical research, and policy attention.

## Figures and Tables

**Table 1:** Demographics of 888 participants in the September 2001 questionnaire survey.

Characteristic	Proportion or measure
Female (%)	59
Age [years (mean ± SD)]	46 ± 9
Race (%)	
White	74
Black	19
Hispanic	6
Building occupancy [years (mean ± SD)]	6 ± 2
Current smoker (%)	14
Never smoker (%)	62

**Table 2:** Comparison of health outcomes prevalences with NHANES III (NCHS 1996).

Standardized questions	Building prevalence [% (*n*)]	Prevalence ratio	95% CI
Asthma ever	17.7 (143/810)	2.2	1.9–2.6
Asthma current	12.8 (103/804)	2.4	2.0–3.0
Wheezing or whistling in your chest in the last 12 months	35.9 (291/811)	2.5	2.2–2.8
Stuffy, itchy, or runny nose in the last 12 months.*^a^*	79.3 (643/811)	1.5	1.4–1.6
Watery, itchy eyes in the last 12 months	63.4 (510/804)	1.6	1.4–1.7
Wheezing, nose, or eye symptoms better on days off work	72.1 (468/649)	3.4	3.1–3.7

The prevalence ratios were adjusted for age, sex, race, and smoking category; missing information on these characteristics led to comparisons based on fewer than the total 888 participants.

a Our question included sneezing.

**Table 3:** The prevalence of work-related lower respiratory symptoms that occurred frequently in the previous 4 weeks, compared to U.S. office workers.

	Prevalence (%)	Prevalence ratio*^a^*	95% CI
Wheezing	6.9	2.9	2.2–3.7
Coughing attacks	14.8	2.7	2.3–3.2
Chest tightness	11.3	4.7	3.8–5.7
Shortness of breath	9.6	4.6	3.7–5.7

a The prevalence of the 888 study participants compared with results from 41 nonproblem buildings (Apte et al. 2000).

**Table 4:** Mean number of lower respiratory symptoms and prevalence of work-related symptoms in the last 4 weeks by asthma status and onset period.

	Postoccupancy-onset asthma	Preoccupancy adult-onset asthma	Childhood-onset asthma	No reported asthma
Number of lower respiratory symptoms (mean ± SD)*^a^*	1.7 ± 1.6*^A^*	1.1 ± 1.3*^B^*	0.7 ± 1.3*^B,C^*	0.5 ± 0.9*^C^*
Work-related lower respiratory symptoms [*n* (%)]	34/66 (52)**	15/37 (41)	8/30 (27)	169/731 (23)

Lower respiratory symptoms include wheeze, cough, chest tightness, and shortness of breath.

a Means with the same letter are not significantly different at α = 0.05, using Duncan’s multiple-range test.

** Cochran-Armitage trend test *p* < 0.0001.

**Table 5:** Demographics of June 2002 participants by respiratory symptom status.

	Respiratory case group (*n* = 140)	Fewer symptoms group (*n* = 63)	Comparison group (*n* = 44)
Female (%)**	73	44	59
Age [years (mean ± SD)]	47 ± 8	46 ± 9	46 ± 8
Occupancy duration [years (mean ± SD)]	7 ± 2	7 ± 2	7 ± 2
Current smoker (%)	17	6	9
Never smoker (%)	56	70	70

Due to missing values, age and duration of occupancy in respiratory case group are based on 137 participants. For age, *n* = 62 in the fewer symptoms group and *n* = 42 in the comparison group.

** *p* = 0.0004 by Chi-square test on sex.

**Table 6:** Breathing test results for participants, stratified by symptom status in June 2002.

Variable	Respiratory cases group	Fewer symptoms group	Comparison group
Spirometry testing [% (*n*)]*^a^*			
Abnormal	24 (31/131)*^b^*	13 (8/62)	7 (3/42)
Obstructed or mixed	15 (20/131)	6 (4/62)	7 (3/42)
Restriction (low FVC)	8 (11/131)	6 (4/62)	0 (0/42)
Percent predicted FEV_1_ (mean ± SD)	92 ± 16*^c^*	96 ± 17	103 ± 12
Percent predicted FVC (mean ± SD)	94 ± 14*^d^*	97 ± 16	103 ± 11
Methacholine challenge testing [% (*n*)]			
Abnormal (< 16 mg/mL)	19 (19/99)	20 (10/51)	6 (2/36)
< 4 mg/mL (BHR)	6 (6/99)	8 (4/51)	0 (0/36)
> 4 and < 16 mg/mL (borderline BHR)	13 (13/99)	12 (6/51)	6 (2/36)
Bronchodilator testing positive [% (*n*)]	18 (2/11)	ND	ND
Abnormal methacholine challenge or bronchodilator tests [% (*n*)]	19 (21/110)	20 (10/51)	6 (2/36)
Any abnormal lung function test [% (*n*)]*^e^*	39 (44/114)*^f^*	29 (16/55)	11 (4/37)

ND, not done.

a Two invalid tests by the symptomatic participants were not included.

b Across the row there was a significant Cochran-Armitage trend test (*p* < 0.01); the significant differences by symptom status remained after adjusting for smoking category (Cochran-Mantel-Haenszel test; *p* < 0.05).

c In a linear regression model adjusting for smoking category, there was a significant effect of symptom status (*p* < 0.01); the group meeting the respiratory case definition had a lower mean percent predicted FEV_1_ than either of the other two groups.

d In a linear regression model adjusting for smoking category, there was a significant effect of symptom status (*p* < 0.01); the group that met the respiratory case definition had a lower mean percent predicted FVC than the asymptomatic group.

e Participants who had either a negative spirometry or a negative methacholine/bronchodilator test and who had not done the other tests were excluded.

f Across the row there was a significant Cochran-Armitage trend test (*p* < 0.01); the significant differences by symptom status remained after adjusting for smoking category (Cochran-Mantel-Haenszel test; *p* < 0.01).

**Table 7:** Medication usage and combined medication usage and abnormal lung function [% (n)] stratified by symptom status in June 2002.

	Respiratory cases group *^a^*	Fewer symptoms	Comparison group
Any medication for breathing problems	46 (65/140)	13 (8/63)	2 (1/44)
Oral steroids	21 (29/140)	8 (5/63)	2 (1/44)
Inhaled steroids	19 (27/140)	2 (1/63)	0 (0/44)
Beta-agonists	28 (39/140)	2 (1/63)	0 (0/44)
Positive for any medication for breathing problems or an abnormal lung function test	67 (83/124)	38 (21/55)	11 (4/37)

a Across all rows there were significant Cochran-Armitage trend tests (*p* < 0.01); the significant differences by symptom status remained after adjusting for smoking category (Cochran-Mantel-Haenszel tests; *p* < 0.01).
